# Dose-Response Relationship between Night Work and the Prevalence of Impaired Fasting Glucose: The Korean Worker’s Special Health Examination for Night Workers Cohort

**DOI:** 10.3390/ijerph18041854

**Published:** 2021-02-14

**Authors:** Jae Yong Lee, Ji-Won Lee, Won Seon Choi, Jun-Pyo Myong

**Affiliations:** 1 Department of Occupational and Environmental Medicine, Seoul St. Mary’s Hospital, College of Medicine, The Catholic University of Korea, Seoul 06591, Korea; monsep86@naver.com; 2 Department of Research for Occupational Health, Institute of Occupation and Environment, Incheon 21417, Korea; celest2120@gmail.com; 3 Suwon Center, Department of Occupational and Environmental Medicine, Korea Medical Institute, Suwon 16571, Korea; cathy302@naver.com

**Keywords:** blood glucose, shift work schedule, worker’s health examination, prediabetic state

## Abstract

Many studies have been conducted regarding the association between night work and diabetes, but the association between impaired fasting glucose (IFG) and night work is still unclear. The aim of this study was to evaluate this association using the Special Health Examination (SHEW) for Korean night workers. Laboratory, questionnaire, and physical examination data were collected for 80,077 manual workers between 2014 and 2016 from Korea Medical Institute, and associations of the data with IFG were evaluated using a multivariate logistic regression model. The odds ratios for IFG among those who worked night shifts for 2~5 years, 5~12 years, and 12 years or over (ref: <2 years) after adjusting for abdominal obesity were 1.14 (0.90–1.45), 1.41 (1.10–1.81), and 1.75 (1.41–2.19), respectively. A dose–response relationship was identified between the duration of night work and the prevalence of IFG (*p* for trend <0.05). A dose relationship remained significant when a subgroup of non-obese participants was analyzed. We identified an association and a dose–response relationship between the number of years of night work and IFG. To prevent the development of diabetes in night workers, we suggest that they should be pre-emptively screened and treated from the stage of IFG.

## 1. Introduction

Diabetes is one of the most significant diseases affecting public health care. It is now highly prevalent and is associated with fatal complications [1]. Globally, diabetes ranks third as the leading risk factor for poor health [2] and is associated with a substantial socio-economic burden in many countries, including a loss of labor productivity [3,4]. Many risk factors for diabetes have been identified, including obesity, family history, ethnicity, and hypertension [5], and recently, an association between night work and diabetes has been identified that is probably related to a disturbance in circadian rhythms [6,7,8].

Medical screening is used to facilitate the early diagnosis and treatment of a target disease. In Korea, workers are obliged to undergo a medical screening program called the “Special Health Examination for Workers (SHEW)” at specific intervals when they are exposed to certain harmful factors that are prescribed by law [9]. Examples of such harmful factors include 180 hazardous chemicals, such as organic compounds and metals, as well as physical factors, such as noise and radiation. In addition, because of concerns regarding the adverse health effects of night work, night work was included as one of the prescribed harmful factors in 2014 [10]. As a result, all workers engaged in night work are legally obliged to undergo a SHEW. The medical conditions that are targeted in the surveillance conducted in night workers are cardiovascular diseases (hypertension, diabetes, hyperlipidemia, and metabolic syndrome), gastrointestinal diseases, breast cancer, and sleep disorders [11]. Measurement of fasting blood glucose is primarily used to diagnose diabetes, but it can also be used to diagnose impaired fasting glucose (IFG), a pre-diabetic finding. Therefore, the SHEW is a useful tool for the early detection of prediabetes in night workers and facilitates early treatment to prevent the development of overt diabetes.

We hypothesized that the prevalence of prediabetes, especially IFG, would increase with the number of years of night work. Also, we hypothesized that the relationship would be independent of obesity. Because the association between night work and IFG has received little attention, we used the screening data for night workers to evaluate the dose–response relationship between number of years of night work and IFG and to identify the most effective stage at which preventative measures against diabetes could be implemented.

## 2. Materials and Methods

### 2.1. SHEW for Night Work

The SHEW is conducted annually for employees working either 8 h of continuous work including the hours from 12 a.m. to 5 a.m. for 6 months (a minimum of four times per month) or ≥60 h on average between 10 p.m. and 6 a.m. the next day for 6 months regardless of the type of night work schedule. It measures waist circumference, blood pressure, fasting blood glucose, and lipid profiles; and records symptoms of gastrointestinal, endocrine, and nervous system disorders as well as the employment history of each individual, their history of night work, and other symptoms. A threshold value is set for each test item, and if the result exceeds this threshold value, or if it is deemed necessary by the doctor who directly interviews the individual, a second assessment is made of the abnormal test item within a month. The threshold for fasting blood glucose is 126 mg/dL, and if this value is exceeded, the measurement is repeated and hemoglobin A1c (HbA1c) is also measured. If the results of the second set of measurements are abnormal, the doctor recommends medical management of the appropriate disease, and if necessary, conducts fitness-for-work testing.

### 2.2. Study Population and Data Collection

Of the individuals who had undergone the SHEW at least once at one of seven Korea Medical Institute (KMI) centers (Gwanghwamum, Yeouido, Gangnam, Suwon, Gwangju, Daegu, or Busan) between 2014 and 2016, those for whom fasting blood glucose data were available were selected as the target group for analysis (*n* = 99,372). Potential participants were excluded if there was missing or erroneous information regarding their medical history, employment history, anthropometry, or lifestyle (*n* = 15,550). Since the period of this study was 3 years for the included participants, some underwent SHEW more than twice. For these participants, the results of the year in which the fasting blood glucose level was the highest were used to classify them into the normal or IFG groups. If the fasting blood glucose was similar over many years and the results for each year were grouped into the same group, the results of the earliest years were used. In this way, a single value was recorded for participants who had undergone multiple examinations, and a final target pool of 83,822 people was obtained. Non-manual workers were excluded from the analysis because there were too few of them (*n* = 72). To focus on IFG, individuals who had already been diagnosed with diabetes or whose blood glucose concentration exceeded the threshold for diabetes at the time of the examination were also excluded from the analysis. The final analysis was conducted on data from 80,077 people (Supplemental Figure S1.)

### 2.3. Ethics

The participants included in this study underwent an SHEW at one of seven KMI centers between 2014 and 2016. The names and social insurance numbers were removed from the records to protect and anonymize the participants, and specific identification numbers were assigned to each. Written informed consent was obtained from all participants. The study was approved by the Institutional Review Board (IRB) of the Catholic University of Korea (KC17RESI0477).

### 2.4. Definition of Covariates

IFG was defined as a fasting blood glucose of 100 mg/dL to 125 mg/dL at the first examination, or a fasting blood glucose of 100 mg/dL to 125 mg/dL and an HbA1c of <6.5% at the second examination. Normality was defined as a fasting blood glucose of <100 mg/dL at the first examination, or a fasting blood glucose of <100 mg/dL and an HbA1c of <6.5% at the second examination. In instances in which HbA1c was not measured at the second examination, the participants were classified on the basis of the fasting blood glucose concentration alone. When the HbA1c was ≥6.5% or when the fasting blood glucose concentration was ≥126 mg/dL at the second examination, diabetes was diagnosed and the individual was excluded from the study. Participants who did not have a history of diabetes, but required a second examination and did not attend, were excluded.

On the basis of the standards for general physical activity in the Korean Adult Physical Activity Guidelines [12], participants who performed moderate exercise (3 to 5.9 metabolic equivalents (METs)) at least three times a week or intense exercise (6 METs and more) at least twice a week during the preceding year were defined as having performed adequate exercise, and if these standards were not met, the participants were regarded as lacking sufficient exercise. The participants were also categorized according to their smoking habits (current smoker, previous smoker, or non-smoker) and drinking habits (>two bottles of Soju (a traditional Korean spirit; one bottle of Soju has a volume of 360 mL and contains 72 g ethanol) per week for <65-year-old men, >one bottle of Soju per week for ≥65-year-old men and <65-year-old women, or >half a bottle of Soju per week for ≥65-year-old women was considered heavy drinking) [13]. The number of years of night work was calculated using the differences between the date of the examination and the date of transfer to current employment position. The durations were compared using the mean values and categorized as quartiles. The potential comorbidities of hypertension and dyslipidemia were assessed using the questionnaire and were defined on the basis of a diagnosis by a doctor. Obesity was defined using two measurements: waist circumference and body mass index (BMI). Abdominal obesity was defined as a waist circumference of ≥90 cm in men and ≥85 cm in women. On the basis of their BMI, participants were classified as normal (<23 kg/m^2^), overweight (23–25 kg/m^2^), obese (25–30 kg/m^2^), or severely obese (≥30 kg/m^2^), according to the World Health Organization’s recommended criteria for Asian people [14].

### 2.5. Statistical Analyses

To compare the characteristics of the IFG group and the normal group, Mann–Whitney and chi-square tests were performed. To validate the collinearity between age and night shift work duration, the Spearman correlation coefficient and Variance inflation factor (VIF) were applied. Logistic regression analysis was used to estimate the *p* for trend of odds ratios for IFG with ascending order of duration of night work as a continuous variable option. Simple and multiple logistic regression analyses were conducted to investigate the association between general characteristics, work characteristics, and the presence of IFG. Odds ratio and 95% confidence intervals were estimated using lowest level of duration of night work as the referent. The two models used all available parameters but Model 1 used overall obesity and Model 2 used abdominal obesity (as these could not be included in the same analysis to avoid collinearity.) Model 1 was adjusted for age, sex, exercise, alcohol intake, smoking, duration of night work, hypertension, dyslipidemia, and overall obesity. Model 2 was adjusted for age, sex, exercise, alcohol intake, smoking, duration of night work, hypertension, dyslipidemia, and abdominal obesity. Besides, additional multiple logistic regression analysis has been performed on subjects over 40 years old, considering the possibility that age acts as a potential confounder of IFG.

A subgroup analysis was also performed to assess the effect of obesity on glucose metabolism, in which participants were stratified according to both abdominal and overall obesity. Data cleaning, data processing, and statistical analyses were performed using SAS 9.4 (SAS Institute, Cary, NC, USA). All statistical tests were two sided using an α level of 0.05.

## 3. Results

Table 1 shows the general and work characteristics of the IFG and normal groups. Of the full cohort, 78,900 (98.53%) were classified as normal and 1177 (1.47%) were classified as having IFG. The IFG group had a higher mean age than the normal group, and the proportion of men was significantly higher. The proportion of participants who performed an adequate amount of exercise was higher in the IFG group than in the normal group, and the proportion of smokers, including ex-smokers, was also significantly higher. The mean duration of night work was also higher in the IFG group (10.5 years), as was the proportion of those who had worked at night for over 12 years (40.78%). In particular, there was a significant positive relationship between the quartile of duration of night work and the prevalence of IFG. The prevalence of both hypertension and dyslipidemia were significantly higher in the IFG group than in the normal group, as was that of obesity.

Table 2 shows the associations between these characteristics. The prevalence of IFG increased with the age of the participants, and this association was significant even after adjustment for other factors, such as lifestyle and work characteristics. In participants of >50 years, the odds ratio (OR) was relatively high, at 9.14 (95% confidence interval [CI] 6.87–12.38) in model 1 and 8.81 (95% CI 6.64–11.92) in model 2. The prevalence of IFG was lower in women than in men, and this difference remained significant after adjustment for other factors. The prevalence of IFG was also higher in the participants who had worked at night for >5 years than in those who had worked at night for <2 years. In addition, although the OR value for the 2–5-year night workers was not significant, the prevalence of IFG increased with the duration of night work. There were also significant associations of overall obesity and abdominal obesity with IFG.

The Spearman correlation coefficient for the relationship between age and duration of night work was evaluated to exclude the possibility that the association identified in this study was due to age, not the duration of night work. The calculated coefficient was −0.09058 (*p* < 0.0001), which implies that the association between these two variables is weak. In addition, the collinearity between the two variables was also assessed by calculating the collinearity indicator, VIF; this was 1.00679, which confirmed low collinearity.

Because the association between IFG and age was so large that it was difficult to interpret the data for the other variables, an additional factor analysis was conducted only for the participants who were ≥40 years old, the age at which the prevalence of IFG significantly increased (Table 3). For the 35,796 participants who were ≥40 years old, the results were similar to those for the full cohort. The mean age of the IFG group was higher than that of the normal group, and men had a higher prevalence of IFG than women. The association between obesity and IFG remained significant in this group, and the tendency for the prevalence of IFG to increase with the duration of night work was also significant.

**Figure 1:**
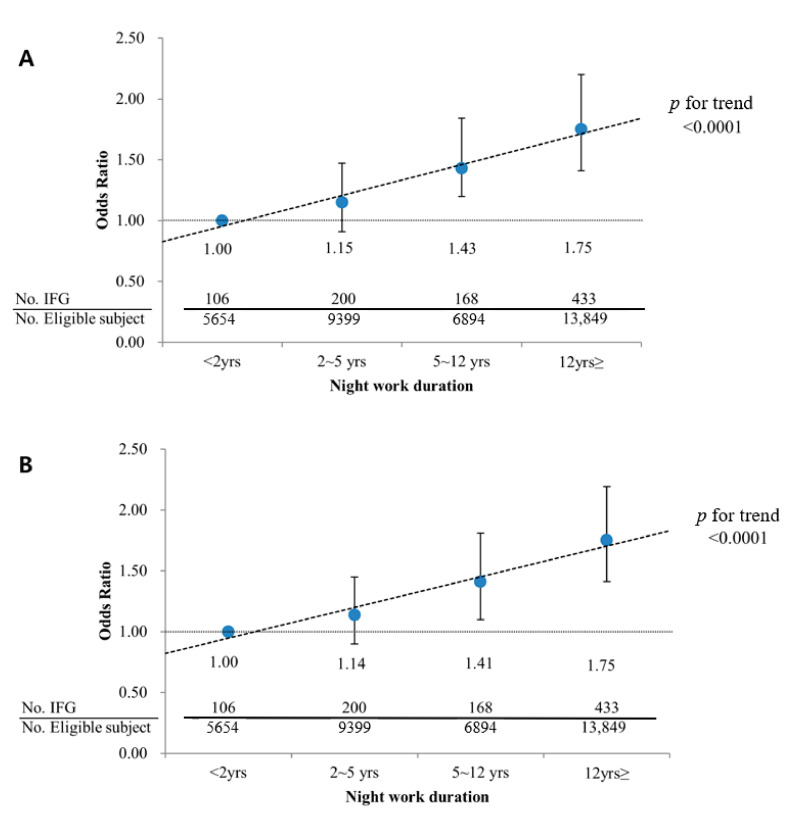
Adjusted odds ratio of IFG (Impaired fasting glucose) according to work duration among subjects over 40 years old. (**A**) Model 1: Adjusted for age, sex, exercise, alcohol habit, smoking, work duration, job type, hypertension, dyslipidemia, obesity (BMI); (**B**) Model 2: Adjusted for age, sex, exercise, alcohol habit, smoking, work duration, job type, hypertension, dyslipidemia, abdominal obesity (waist circumference). Logistic regression analysis was used to estimate the *p* for trend of odds ratios for IFG with ascending order of duration of night work as continuous variable option.

In participants who were >40 years old, the association between IFG and the duration of night work remained significant after other factors were adjusted for, and these results are presented according to the duration of night work in Figure 1. 

The prevalence of IFG increased significantly with the duration of night work (*p* for trend <0.0001 in Model 1, *p* for trend <0.0001 in Model 2). For participants who worked night for 5–12 years, the OR was 1.43 (95% CI 1.20–1.84) in Model 1 and 1.41 (95% CI 1.10–1.81) in Model 2; and for those who worked at night for >12 years, the ORs were 1.75 (95% CI 1.41–2.20) in Model 1 and 1.75 (95% CI 1.41–2.19) in Model 2.

**Figure 2:**
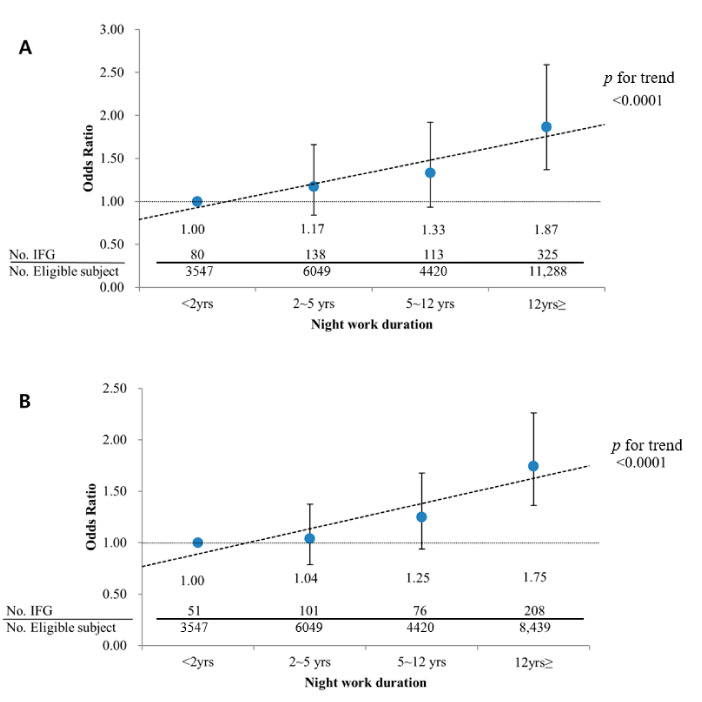
Adjusted odds ratio of IFG (Impaired fasting glucose) according to work duration, stratified by obesity (either waist circumference or BMI, alternatively) among subjects over 40 years old. (**A**) subjects without obesity (BMI); (**B**) subjects without abdominal obesity (waist circumference). Adjusted for age, sex, exercise, alcohol habit, smoking, work duration, job type, hypertension, dyslipidemia. Logistic regression analysis was used to estimate the *p* for trend of odds ratios for IFG with ascending order of duration of night work as continuous variable option.

A subgroup analysis (Figure 2) was also performed for participants who were not obese, because of the well-known association between obesity and diabetes [15,16]. In these participants, there was also a significant association between the duration of night work and IFG. The ORs for participants who had worked at night for >12 years were 1.87 (95% CI 1.37–2.59) in those with BMI as normal and 1.75 (95% CI 1.36–2.26) in those whose waist circumference was normal.

## 4. Discussion

An analysis of medical screening data for 80,077 individuals who engaged in night work identified an association between night work and IFG that was dose-responsive. This association became more significant after correction for obesity and other potential confounding factors, and remained even if the data were stratified according to the age of the participants.

The mechanisms underlying the higher risk of diabetes among night workers have not been fully delineated, but several have been suggested. The first is metabolic disturbance due to circadian misalignment. Mammals, including humans, survive by synchronizing their behavioral cycle with environmental cycles, such as the light-dark cycle, using endogenous circadian systems [17]. However, this synchronization is upset by external factors, such as night work or social jet lag, which have deleterious effects on metabolism and the cardiovascular system [18,19]. Specifically, disruption of the normal circadian rhythm reduces the secretory function of pancreatic β-cells, interferes with the growth and proliferation of the cells, and increases their susceptibility to oxidative stress, all of which together lead to β-cell failure [20]. In addition, long-term night work induces insulin resistance and hyperglycemia by causing an inversion of the circadian pattern of cortisol secretion [18]. All these mechanisms would cause disturbances in insulin-glucose control and increase the risk of diabetes. A further problem is that night workers often change their bedtime [21], which reduces both the duration of and quality of sleep [22,23]. Such sleep disturbance further disrupts key circadian rhythms, causing vicious cycles of induction and exacerbation of insulin resistance, and reciprocal changes in leptin and ghrelin secretion [24], which impair glycemic control [25]. Finally, stress may also explain the higher risk of diabetes among shift workers [26] and affect other relevant aspects of behavior, such as smoking or eating habits, which may also predispose toward diabetes [27].

Obesity is an important potential mediator of the association between night work and IFG. According to the results of previous studies, night work is positively associated with weight gain [28,29], which is a major risk factor for diabetes, and in addition, it increases appetite and adiposity [30,31]. Specifically, BMI has been shown to at least partly mediate the association between night work and diabetes [27]. Therefore, because obesity could be responsible for the association between night work and IFG, we adjusted the data for both overall and abdominal obesity, and performed a subgroup analysis. However, even after adjustment for obesity, the dose–response relationship between the duration of night work and IFG remained significant. Consistent results were also found in the subgroup analysis that was conducted on non-obese workers, which implies that even non-obese individuals are at a greater risk of developing diabetes in the future if they do shift work. A previous study conducted in Japan [32] showed that workers with prediabetes, defined using IFG and impaired glucose tolerance, were at a higher risk of developing diabetes if they worked at night than if they worked during the day. Therefore, even for non-obese workers, the risks of developing IFG and diabetes from prediabetes showed an increase with the duration of night work.

In addition, the dose–response relationship was analyzed by considering abdominal obesity and overall obesity simultaneously. The significance of increasing the prevalence of IFG according to the duration of night work was identified in the normal group without both abdominal and overall obesity (*p* for trend <0.0001), but the significance disappeared in those with only abdominal obesity, only overall obesity, or with both simultaneously. (Supplemental Figure S2). According to the results of a previous study [27], obesity acts partly as a factor mediating the association between night work and diabetes. However, in the present study, we found that it was, at best, a weak or null mediator of the association between night work and IFG. Considering both of these results, if individuals with diabetes had not been excluded from the study, it may be possible that a significant trend between duration of night work and prevalence of IFG would have been identified in those with only abdominal obesity, only overall obesity, or with both simultaneously.

As previously shown, age is a major risk factor for glucose impairment [5,33]. The correlation or multicollinearity between age and night work duration was shown in the present study, however, age itself is a major risk factor for IFG. Therefore, in the present study, because age had a larger effect on the prevalence of IFG than other factors, a subgroup analysis was performed on participants in different age groups (Supplemental Table S1). The results of this analysis also showed a dose–response relationship between IFG and night work, which confirms that the effect of night work itself is significant and separate from that of age.

Low estrogen levels can lead to insulin resistance and diabetes [34]. Through this mechanism, female night workers might have a relatively lower risk of diabetes than male night workers, which was concordant with the results of this study.

### 4.1. Limitations

This was a cross-sectional study, and therefore a causal relationship between night work and IFG cannot be inferred. In addition, a caution should be followed in interpreting odds ratios as they are dependent on other parts of the model being fixed/adjusted for in the present study. However, we were able to identify an association between night work and IFG, and at the same time we were able to identify a biological gradient (dose–response relationship), one of the Bradford Hill criteria for determining a causal relationship.

Despite the legal requirement, some companies did not participate in the screening scheme described. Poorly financed workplaces tend not to receive health examinations for economic reasons, and the health status of workers in such workplaces may be relatively poor [35]. Working for such companies is associated with a greater cumulative risk of night work, and taking this into account, the prevalence of IFG revealed in the present study may have been underestimated.

Since the results were obtained using a questionnaire that was originally designed for the purpose of health examination, some information about night work was missing. According to previous study [36], the risk of diabetes differs depending on the type of night work schedule, such as fixed type and rotation type; however, this could not be assessed because information on the type of night work schedule could not be obtained using this questionnaire. In addition, since the definition of work duration was the number of years spent doing night work in the current workplace, it was not possible to determine the effect of life-time night work, which might have revealed the cumulative contribution of night work to IFG and diabetes. Lastly, in addition to the covariates suggested in this study, other factors that might affect night work and fasting blood glucose levels were not sufficiently obtained and controlled. In the future study, these characteristics should also be addressed.

The “healthy worker effect” is also present in night workers [37]. Assuming that this phenomenon affected the night workers in the present study, the prevalence of IFG in the “survivors” would have underestimated the actual prevalence of IFG associated with night work. In addition, we used workers that had worked 0 to 2 years at night as the reference group, which is not strictly speaking a good control group. If a group of workers who had never worked at night could have been used as the reference group, a clearer association and trend might have been forthcoming. In the present study, this was not possible, because only night workers were permitted to participate in the screening program by law, but the trend should be verified in a future study that includes a group of workers who do not perform night work.

### 4.2. Strengths

Many previous studies [38,39] have shown a higher risk of diabetes in night workers, but this study is the first, to our knowledge, to focus on IFG, a type of prediabetes. The fact that prediabetes, which is regarded as an important finding during screening, is prevalent in night workers, means that this group should be targeted for the early diagnosis and treatment of diabetes risk. Although the study subjects were not randomly extracted, the study has the strength that the subjects were from seven nationally distributed branch centers, not a single institution, and large numbers of workers were examined.

## 5. Conclusions

In the present study, we have demonstrated an association between night work and IFG, which was dose-responsive. Those dose–response relationship is also shown among non-obese male manual shift workers. For workers’ health, legislation is required to minimize night work, and in industries where night work is indispensable, pre-emptive screening for IFG should be performed to facilitate the prevention of diabetes. In addition, the early intervention, such as periodic follow-up, might be necessary for a potential vulnerable working population, such as those who already have a family history of diabetes.

## Figures and Tables

**Table 1:** General characteristics of participants who underwent the SHEW.

	Normal, *N* (%) (*N* = 78,900)	IFG, *N* (%) (*N* = 1177)	*p*-Value
Age			
38.8 ± 11.8 (years)	38.6 ± 11.8	47.7 ± 10.4	<0.0001
<20 s	21,608 (27.39)	55 (4.67)	<0.0001
30 s	22,403 (28.39)	215 (18.27)	
40 s	18,617 (23.60)	361 (30.67)	
≥50 s	16,272 (20.62)	546 (46.39)	
Sex			<0.0001
Male	59,422 (75.31)	1045 (88.79)	
Female	19,478 (24.69)	132 (11.21)	
Exercise			0.0027
Adequate	35,557 (45.07)	582 (49.45)	
Lack of exercise	43,343 (54.93)	595 (50.55)	
Alcohol drinking			0.1569
Adequate	56,383 (71.46)	819 (69.58)	
Heavy	22,517 (28.54)	358 (30.42)	
Smoking			<0.0001
Non-smoker	34,684 (43.96)	390 (33.14)	
Ex-smoker	16,218 (20.56)	396 (33.64)	
Current-smoker	27,998 (35.49)	391 (33.22)	
Duration of night work			
6.1 ± 7.7 (years)	6.0 ± 7.7	10.5 ± 10.0	<0.0001
<2 years	16,757 (21.24)	146 (12.40)	<0.0001
2–5 years	27,823 (35.26)	281 (23.87)	
5–12 years	17,452 (22.12)	270 (22.94)	
≥12 years	16,868 (21.38)	480 (40.78)	
History of hypertension			<0.0001
No	73,870 (93.62)	952 (80.88)	
Yes	5030 (6.38)	225 (19.12)	
History of dyslipidemia			0.0046
No	77,698 (98.48)	1147 (97.45)	
Yes	1202 (1.52)	30 (2.55)	
BMI			<0.0001
<23 kg/m^2^	32,371 (41.03)	250 (21.24)	
23–24.9 kg/m^2^	18,912 (23.97)	278 (23.62)	
25–29.9 kg/m^2^	23,343 (29.59)	535 (45.45)	
≥30 kg/m^2^	4274 (5.42)	114 (9.69)	
Abdominal obesity			* < 0.0001
No	65,123 (82.54)	814 (69.16)	
Yes	13,777 (17.46)	363 (30.84)	

IFG: impaired fasting glucose; BMI: body mass index; Age and duration of night work were described as mean ± SD (Standard deviation). Statistics are presented as mean ± SD (Standard deviation) or number (%). * *p*-value < 0.05.

**Table 2:** Associations between the general characteristics of the participants and IFG.

	Crude		Model 1 ^†^		Model 2 ^‡^	
	OR	95% CI	OR	95% CI	OR	95% CI
Duration of night work						
<2 years	1.00		1.00		1.00	
2~5 years	1.16	0.95–1.42	1.14	0.93–1.40	1.12	0.92–1.38
5~12years	1.78 *	1.45–2.18	1.51 *	1.23–1.86	1.49 *	1.21–1.84
≥12 years	3.27 *	2.72–3.95	1.75 *	1.44–2.12	1.74 *	1.44–2.12
Age						
≤20 s	1.00		1.00		1.00	
30 s	3.77 *	2.83–5.12	2.67 *	1.99–3.65	2.77 *	2.06–3.79
40 s	7.62 *	5.79–10.23	4.91 *	3.67–6.68	4.96 *	3.72–6.75
≥50 s	13.18 *	10.08–17.60	9.14 *	6.87–12.38	8.81 *	6.64–11.92
Sex						
Male	1.00		1.00		1.00	
Female	0.39 *	0.32–-0.46	0.53 *	0.43–0.65	0.50 *	0.40–0.61
Exercise						
Adequate	1.00		1.00		1.00	
Lack of exercise	0.84 *	0.75–0.94	1.15 *	1.03–1.30	1.12	0.99–1.26
Drinking						
Adequate	1.00		1.00		1.00	
Heavy	1.10	0.97–1.24	1.14	1.00–1.30	1.16	1.02–1.32
Smoking						
Non-smoker	1.00		1.00		1.00	
Ex-smoker	2.17 *	1.89–2.50	1.09	0.93–1.28	1.10	0.94–1.29
Current-smoker	1.24 *	1.08–1.43	0.96	0.82–1.13	0.95	0.81–1.11
History of hypertension						
Normal	1.00		1.00		1.00	
Hypertension	3.47 *	2.99–4.02	1.58 *	1.34–1.84	1.67 *	1.42–1.95
History of dyslipidemia						
Normal	1.00		1.00		1.00	
Dyslipidemia	1.69 *	1.15–2.39	0.84	0.57–1.20	0.84	0.57–1.20
BMI						
Normal	1.00		1.00			
Overweight	1.90 *	1.60–2.26	1.37 *	1.15–1.63		
Obesity	2.97 *	2.56–3.46	2.10 *	1.80–2.45		
Severe obesity	3.45 *	2.75–4.31	3.62 *	2.87–4.54		
Abdominal obesity						
Normal	1.00				1.00	
Abdominal obesity	2.11 *	1.86–2.39			1.79 *	1.57–2.04

* *p*-value < 0.05. ^†^ Adjusted for age, sex, exercise, alcohol intake, smoking, duration of night work, hypertension, dyslipidemia, and overall obesity (BMI). ^‡^ Adjusted for age, sex, exercise, alcohol intake, smoking, duration of night work, hypertension, dyslipidemia, and abdominal obesity (waist circumference).

**Table 3:** General characteristics of participants aged >40 years and associations between these characteristics and IFG.

	Normal, *N* (%)	IFG, *N* (%)	Crude		Model 1 ^†^		Model 2 ^‡^	
			OR	95% CI	OR	95% CI	OR	95% CI
Duration of night work *								
9.5 ± 9.6 (years)	9.5 ± 9.6	12.0 ± 10.6						
<2 years	5548 (15.90)	106 (11.69)	1.00		1.00		1.00	
2~5 years	9199 (26.37)	200 (22.05)	1.14	0.90–1.45	1.15	0.91–1.47	1.14	0.90–1.45
5~12years	6726 (19.28)	168 (18.52)	1.31 *	1.02–1.68	1.43 *	1.20–1.84	1.41 *	1.10–1.81
≥12 years	13,416 (38.45)	433 (47.74)	1.69 *	1.37–2.10	1.75 *	1.41–2.20	1.75 *	1.41–2.19
Age * (years)								
50.0 ± 7.1	49.9 ± 7.1	51.9 ± 7.4	1.04 *	1.03–1.05	1.04 *	1.03–1.05	1.04 *	1.03–1.05
Sex *								
Male	27,401 (78.54)	815 (89.86)	1.00		1.00		1.00	
Female	7488 (21.46)	92 (10.14)	0.41 *	0.33–0.51	0.47 *	0.37–0.61	0.46 *	0.35–0.59
Exercise								
Adequate	18,589 (53.28)	496 (54.69)	1.00		1.00		1.00	
Lack of exercise	16,300 (46.72)	411 (45.31)	0.95	0.83–1.08	1.07	0.94–1.23	1.05	0.92–1.20
Drinking *								
Adequate	26,858 (76.98)	653 (72.00)	1.00		1.00		1.00	
Heavy	8031 (23.02)	254 (28.00)	1.30 *	1.12–1.51	1.16	0.99–1.35	1.17 *	1.00–1.37
Smoking *								
Non-smoker	14,190 (40.67)	300 (33.08)	1.00		1.00		1.00	
Ex-smoker	10,283 (29.47)	339 (37.38)	1.56 *	1.33–1.83	1.03	0.86–1.23	1.04	0.88–1.25
Current-smoker	10,416 (29.85)	268 (29.55)	1.22 *	1.03–1.44	0.94	0.78–1.14	0.92	0.77–1.11
History of hypertension *								
Normal	30,461 (87.31)	693 (76.41)	1.00		1.00		1.00	
Hypertension	4428 (12.69)	214 (23.59)	2.13 *	1.81–2.48	1.53 *	1.29–1.81	1.64 *	1.38–1.93
History of dyslipidemia								
Normal	33,896 (97.15)	880 (97.02)	1.00		1.00		1.00	
Dyslipidemia	993 (2.85)	27 (2.98)	1.05	0.69–1.51	0.82	0.54–1.19	0.83	0.54–1.20
BMI *								
Normal	12,231 (35.06)	194 (21.39)	1.00		1.00		1.00	
Overweight	9788 (28.05)	242 (26.68)	1.56 *	1.29–1.89	1.40 *	1.16–1.70		
Obesity	11,699 (33.53)	415 (45.76)	2.24 *	1.89–2.66	1.95 *	1.64–2.33		
Severe obesity	1171 (3.36)	56 (6.17)	3.02 *	2.21–4.05	2.88 *	2.10–3.90		
Abdominal obesity *								
Normal	28,600 (81.97)	656 (72.33)	1.00		1.00		1.00	
Abdominal obesity	6289 (18.03)	251 (27.67)	1.74 *	1.50–2.02			1.53 *	1.31–1.78
Total number	34,889 (97.47)	907 (2.53)						

* *p*-value < 0.05; Age and duration of night work were described as mean ± SD (Standard deviation); Boldface data have *p*-values < 0.05 in logistic regression analysis. ^†^ Model1: Adjusted for age, sex, exercise, alcohol intake, smoking, duration of night work, hypertension, dyslipidemia, and overall obesity (BMI). ^‡^ Model2: Adjusted for age, sex, exercise, alcohol intake, smoking, duration of night work, hypertension, dyslipidemia, and abdominal obesity (waist circumference).

## Data Availability

Data available on request due to restrictions privacy or ethical. The data presented in this study are available on request from the corresponding author.

## Supplementary Materials

The following are available online at https://www.mdpi.com/1660-4601/18/4/1854/s1. Figure S1. Adjusted odds ratio of IFG (Impaired fasting glucose) according to duration of night work, stratified by obesity (BMI & waist circumference) among subjects over 40 years old. Figure S2. Adjusted odds ratio of IFG (Impaired fasting glucose) according to work duration, stratified by obesity (BMI & waist circumference) among subjects over 40 years old. Table S1. Trend for IFG according to night work duration and age group.
