# Supplementary material for: Dose-Response Relationship between Night Work and the Prevalence of Impaired Fasting Glucose: The Korean Worker’s Special Health Examination for Night Workers Cohort
Source: Int J Environ Res Public Health. 2021 Feb 14;18(4):1854. doi: 10.3390/ijerph18041854 (PMC7918366; doi:10.3390/ijerph18041854)
Supplement: Supplementary file 1 [file ijerph-18-01854-s001.zip › Supplemental Table 1.docx]

**Supplemental Table 1.** Trend for IFG according to night work duration and age group

|  | Model 1* | | | | Model 2† | | | | | | P for trend | | | |  |
| --- | --- | --- | --- | --- | --- | --- | --- | --- | --- | --- | --- | --- | --- | --- | --- |
|  | OR | | 95% CI | | OR | | | | 95% CI | |  | | | |  |
| Work duration-20s & 30s |  | |  | |  | | | |  | | <.0001 | | | |  |
| <2 years | 1.00 | |  | | 1.00 | | | |  | |  | | | |  |
| 2~5 years | 1.17 | | 0.81–1.73 | | 1.17 | | | | 0.80–1.72 | |  | | | |  |
| 5~12years | **2.39** | | **1.67–3.50** | | **2.40** | | | | **1.67–3.50** | |  | | | |  |
| ≥12 years | **3.63** | | **2.37–5.59** | | **3.60** | | | | **2.35–5.54** | |  | | | |  |
| Work duration-40s | |  | |  | | | |  | | |  | | <.0001 | | |
| <2 years | 1.00 | |  | | | 1.00 | | |  | | |  | |  |  |
| 2~5 years | 1.11 | | 0.74–1.71 | | | 1.09 | | | 0.72–1.67 | | |  | |  |  |
| 5~12years | 1.34 | | 0.89–2.07 | | | 1.31 | | | 0.87–2.01 | | |  | |  |  |
| ≥12 years | **1.73** | | **1.21–2.55** | | | **1.68** | | | **1.18–2.47** | | |  | |  |  |
| Work duration-50s | |  | |  | | | |  | | |  | | <.0001 | | |
| <2 years | 1.00 | |  | | | | 1.00 | | |  | |  | |  |  |
| 2~5 years | 1.19 | | 0.89–1.60 | | | | 1.18 | | | 0.89–1.59 | |  | |  |  |
| 5~12years | **1.51** | | **1.11–2.05** | | | | **1.49** | | | **1.10–2.03** | |  | |  |  |
| ≥12 years | **1.59** | | **1.22–2.10** | | | | **1.61** | | | **1.24–2.13** | |  | |  |  |

*Adjusted for age, sex, exercise, alcohol intake, smoking, duration of night work, hypertension, dyslipidemia, and overall obesity (BMI).

†Adjusted for age, sex, exercise, alcohol intake, smoking, duration of night work, hypertension, dyslipidemia, and abdominal obesity (waist circumference)
