# Supplementary figures and images for: Dose-Response Relationship between Night Work and the Prevalence of Impaired Fasting Glucose: The Korean Worker’s Special Health Examination for Night Workers Cohort
Source: Int J Environ Res Public Health. 2021 Feb 14;18(4):1854. doi: 10.3390/ijerph18041854 (PMC7918366; doi:10.3390/ijerph18041854)

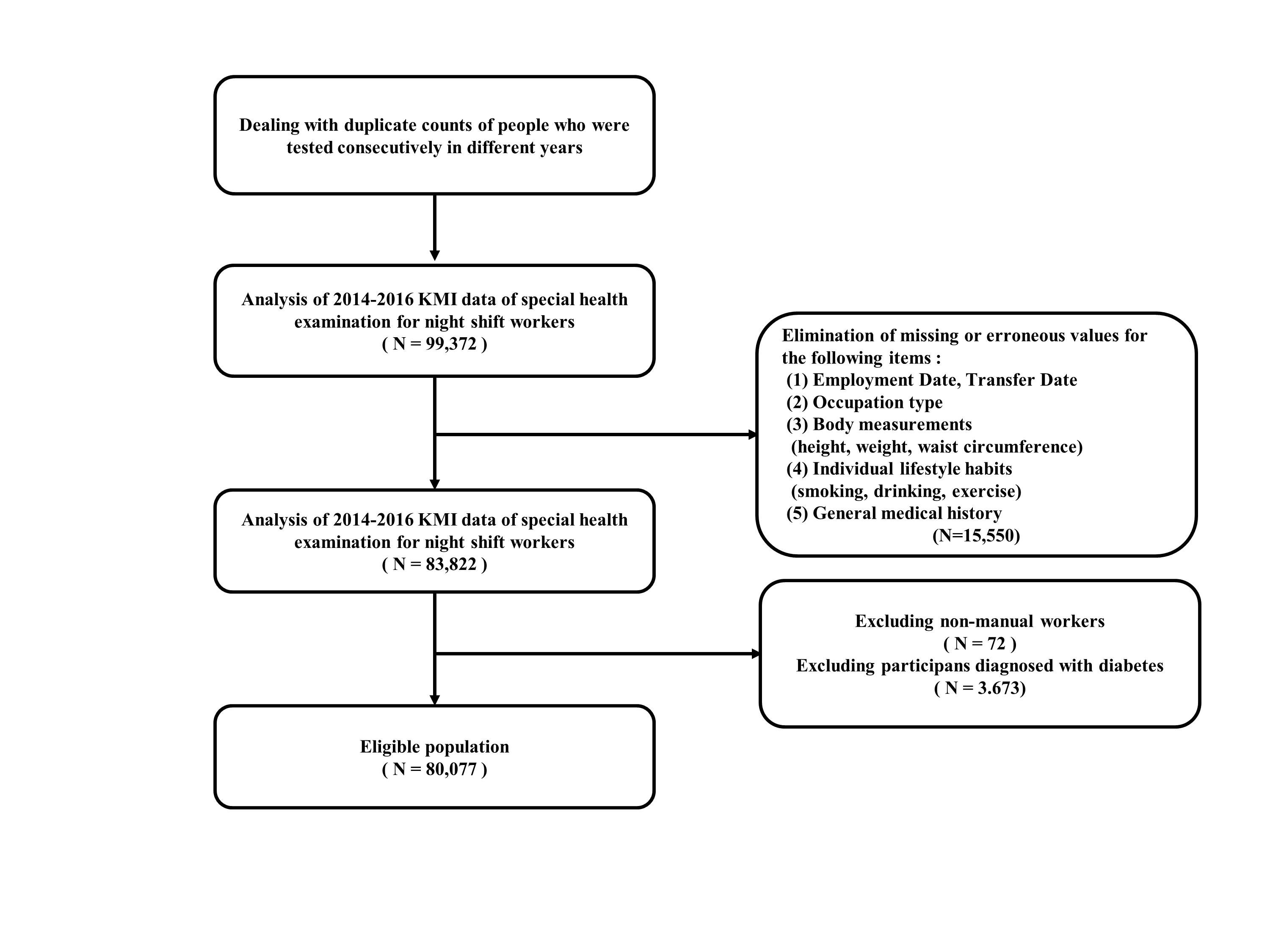

Supplement: Supplementary file 1 [file ijerph-18-01854-s001.zip › Figure S1.tif]

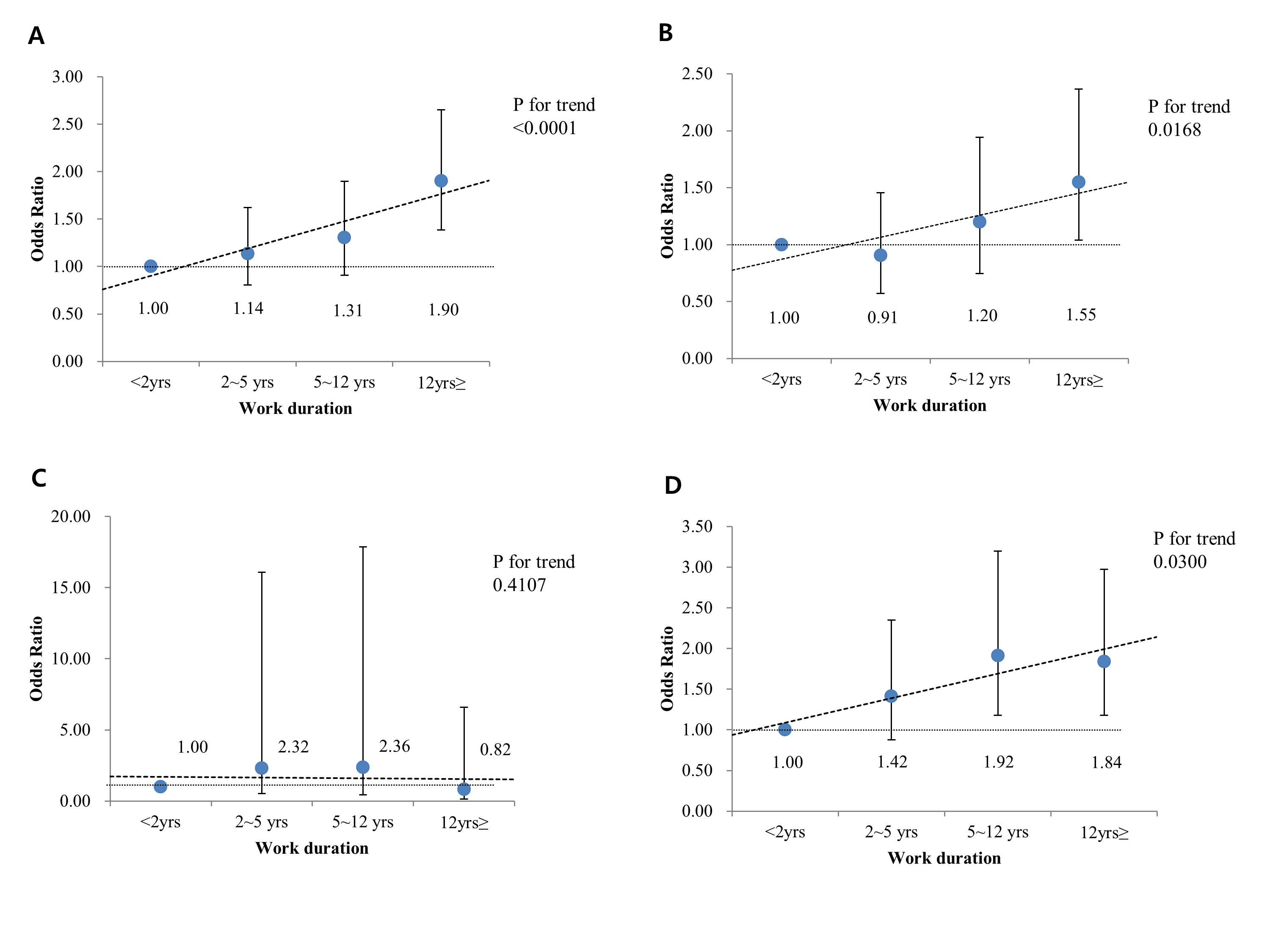

Supplement: Supplementary file 1 [file ijerph-18-01854-s001.zip › Figure S2.tif]
